# Enhancement of Anti-Melanogenic Activity of *Pinctada martensii* Nacre-Derived Peptide Through *C*-Terminal Amidation: Mechanism Investigation in B16F10 Cells

**DOI:** 10.3390/md24090303

**Published:** 2026-08-30

**Authors:** Haisheng Lin, Ruonan Chang, Fei Li, Jialong Gao, Zhongqin Chen, Yuanwei Liang, Wenhong Cao, Huina Zheng

**Affiliations:** 1 National Research and Development Branch Center for Shellfish Processing (Zhanjiang), Guangdong Provincial Key Laboratory of Aquatic Products Processing and Safety, Guangdong Provincial Engineering Technology Research Center of Seafood, Guangdong Province Engineering Laboratory for Marine Biological Products, College of Food Science and Technology, Guangdong Ocean University, Zhanjiang 524088, China; haishenglin@163.com (H.L.);; 2 College of Chemistry and Environmental Science, Guangdong Ocean University, Zhanjiang 524088, China; 3 Shenzhen Institute of Guangdong Ocean University, Shenzhen 518108, China

**Keywords:** *Pinctada martensii*, melanin, chemical modification, molecular mechanism

## Abstract

Excessive melanin accumulation induced by UV exposure and hormonal changes poses significant challenges to skin health, necessitating safe and effective tyrosinase inhibitors. Pearl and nacre powder have long been utilized in traditional Chinese medicine for skin whitening. Our previous study identified a tyrosinase inhibitory peptide (Ala-His-Tyr-Tyr-Asp) from *Pinctada martensii* nacre in silico; however, chemical modification strategies for enhancing bioactive peptide efficacy remain underexplored. The present study investigated the structure-activity relationship of NP-TIP-1 (Tyrosinase Inhibitory Peptide of Nacre Powder-1) through three chemical modifications (*C*-terminal amidation, *N*-terminal acetylation, and *N*-terminal palmitoylation). *C*-terminal amidation significantly improved tyrosinase inhibitory potency, reducing the IC50 value from 2.012 ± 0.088 mM to 0.979 ± 0.028 mM, while retaining thermal stability and copper ion chelating capacity comparable to the native peptide. In B16F10 melanoma cells, NP-TIP-1-NH_2_ (C-terminally amidated NP-TIP-1) exhibited no cytotoxicity at concentrations up to 600 μM and concentration-dependently suppressed intracellular tyrosinase activity and melanin content. Mechanistic investigation revealed that NP-TIP-1-NH_2_ treatment downregulates the mRNA and protein expression of TYR and TRP-1 without affecting MITF (Microphthalmia-associated Transcription Factor) or TRP-2, suggesting a potential regulatory mechanism independent of MITF-mediated transcriptional control. Non-targeted metabolomics further identified nucleotide metabolism, purine metabolism, and glycine/serine/threonine metabolism as the most significantly perturbed pathways, with cytosine and GMP downregulated while guanosine and guanine were upregulated. This study establishes NP-TIP-1-NH_2_ as a promising candidate for multifunctional food preservatives and nutraceuticals, providing a strategic framework for *C*-terminal amidation as a structure-activity optimization approach for marine-derived anti-melanogenic peptides.

## 1. Introduction

Melanin is a widely distributed phenolic polymer found in both plants and animals [1]. It is the primary determinant of skin and hair color, as it absorbs ultraviolet rays, reduces DNA damage, and lowers the risk of skin cancer [2]. However, excessive melanin production can lead to an accumulation of pigment in the superficial layers of the skin, resulting in various skin conditions such as dark spots and brown patches. This overproduction can also accelerate skin aging and potentially contribute to the development of melanoma and other skin cancers. Therefore, inhibiting melanin overproduction can aid in achieving skin whitening and preventing melanin-related diseases.

Melanin synthesis is the process by which melanocytes produce pigment within melanosomes and is primarily composed of eumelanin and pheomelanin. This biosynthesis involves a series of complex enzymatic reactions, including those catalyzed by tyrosinase (TYR), tyrosinase-related protein 1 (TRP-1), and tyrosinase-related protein 2 (TRP-2) [3]. Tyrosinase is a copper-containing metalloenzyme and serves as the key regulatory enzyme in melanogenesis [4]. It catalyzes the oxidation of L-tyrosine to dopaquinone, thereby initiating melanin synthesis. In the presence of cysteine, dopaquinone preferentially undergoes a rapid reaction with cysteine to form cysteinyldopa, which then undergoes a series of oxidation, cyclization, and rearrangement reactions, ultimately polymerizing to produce pheomelanin. Once cysteine is depleted, dopaquinone undergoes intramolecular cyclization and oxidation to generate dopachrome. Dopachrome undergoes decarboxylation to yield 5,6-dihydroxyindole (DHI), which is further transformed into the melanin intermediate 5,6-indolequinone (IQ) through oxidative rearrangement. Meanwhile, dopachrome can be converted into 5,6-dihydroxyindole-2-carboxylic acid (DHICA) under the action of TRP-2 and subsequently oxidized by TRP-1 to form indole-5,6-quinone-2-carboxylic acid (ICAQ). IQ and ICAQ polymerize to form eumelanin. Therefore, the inhibition of tyrosinase activity and antioxidant effects are particularly important in regulating melanin production [5,6].

There are primarily two pathways for inhibiting tyrosinase: the first involves tyrosinase inhibitors directly targeting the active site of the enzyme; the second involves indirectly influencing tyrosinase levels by regulating the antioxidant enzyme system and antioxidant metabolites [7]. Currently, proven effective whitening agents such as kojic acid and its derivatives, arbutin, and others have demonstrated significant inhibitory effects on tyrosinase. However, these ingredients may also lead to various side effects. Despite the widespread use of existing whitening products, there remains a considerable risk of allergic reactions and other adverse effects among users [8]. Consequently, there is growing interest in the development of safe and stable whitening formulations.

Researchers screened a series of tyrosinase-inhibiting peptides derived from the gelatin of sea cucumber body walls [9], as well as from grass carp [10], mackerel meat [11], pearl oyster meat [12], and other sources. Research indicates that the primary components of nacre powder closely resemble those of pearl powder and exhibit a range of bioactive properties. Among the functional activities of pearl powder that have been validated by modern medicine are antibacterial, anti-photoaging, antioxidant, whitening, and blood pressure-lowering effects; however, the specific components and factors responsible for the whitening effect remain unclear. Following pearl harvesting, the *Pinctada martensii* generates a substantial amount of byproducts, which include not only the pearl meat but also a significant quantity of shell material. Currently, the utilization rate of the shell is approximately 30%, with primary applications including decoration, coatings, and animal feed. Our previous study identified a tyrosinase inhibitory peptide (Ala-His-Tyr-Tyr-Asp) from *Pinctada martensii* nacre through in silico screening, which exhibited mixed-type inhibition against tyrosinase and reduced melanin content in B16F10 cells via direct enzyme inhibition and antioxidant activity [13]. However, the moderate potency and structural limitations of the native peptide necessitate further optimization to enhance its applicability in food and pharmaceutical systems; thus, chemical modification strategies for improving bioactive peptide efficacy remain underexplored.

Natural bioactive peptides often exhibit specific functional activities; however, they frequently present challenges such as poor water solubility, limited permeability, and unstable structures. Consequently, modifying bioactive peptides through chemical methods can improve their functional activities and physicochemical properties while ensuring a non-toxic dosage [14]. The chemical modification of antimicrobial peptides has become increasingly common, with the primary modification methods for active peptides including *C*-terminal modification, *N*-terminal modification, glycosylation, cyclization, side chain modification, and others. Among these, glycosylation is currently the most widely applied technique for antimicrobial peptides [15]. Research has shown that glycosylation can enhance the efficacy of antimicrobial peptides and aid in the design of novel peptide-based delivery systems. For instance, *O*-glycosylation not only influences the cellular uptake of peptides but also interacts with their intracellular target, ribosomes [16]. Other modifications, such as the cyclization of umami peptides, can induce electrostatic rearrangements that lead to variations in umami perception [17]. Research on the effects of *N*-terminal and *C*-terminal modifications on peptide self-assembly has demonstrated that *N*-terminal modifications enhance structural stability, confer anti-inflammatory properties, and improve wound healing capacity; furthermore, *C*-terminal modification with lysine significantly boosts antimicrobial properties [18].

In this study, the pentapeptide (Ala-His-Tyr-Tyr-Asp), previously identified from the nacre layer of *Pinctada martensii*, was subjected to three chemical modifications (*C*-terminal amidation, *N*-terminal acetylation, and *N*-terminal palmitoylation) to enhance its tyrosinase inhibitory potency and structural stability. The structure-activity relationships of the modified peptides were systematically evaluated through in vitro enzymatic assays, biophysical characterization, and cellular investigations in B16F10 melanoma cells, with particular emphasis on elucidating the underlying mechanisms involving gene expression, protein regulation, and metabolic pathway modulation.

## 2. Results and Discussion

### 2.1. Verification of the In Vitro Tyrosinase Activity and Antioxidant Activity of Modified Peptides

**Figure 1:**
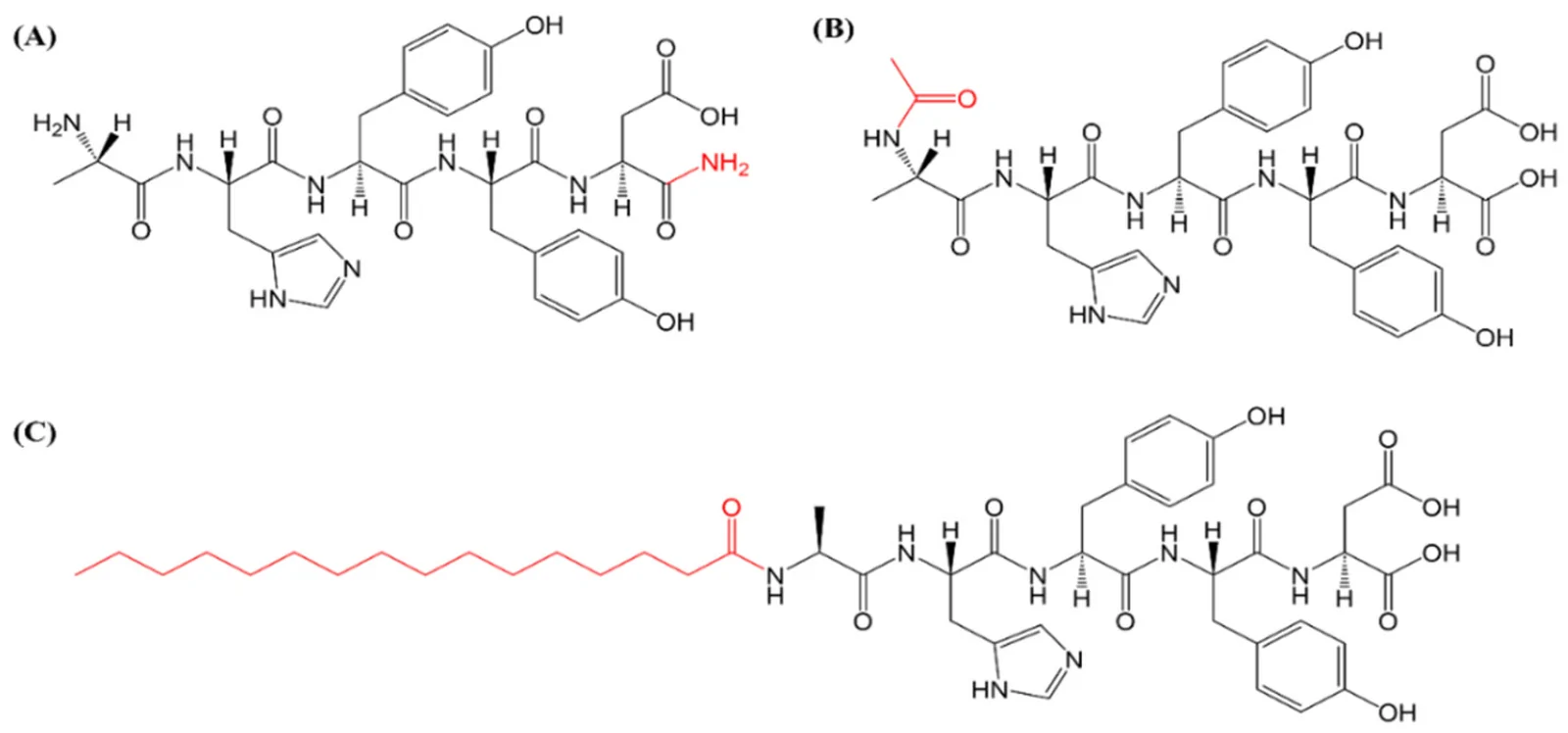
Chemical structure diagram of modified peptides. The red-colored regions indicate the chemical modification sites of the peptide. (**A**): NP-TIP-1-NH_2_; (**B**): Ac-NP-TIP-1; (**C**): Pal-NP-TIP-1.

**Figure 2:**
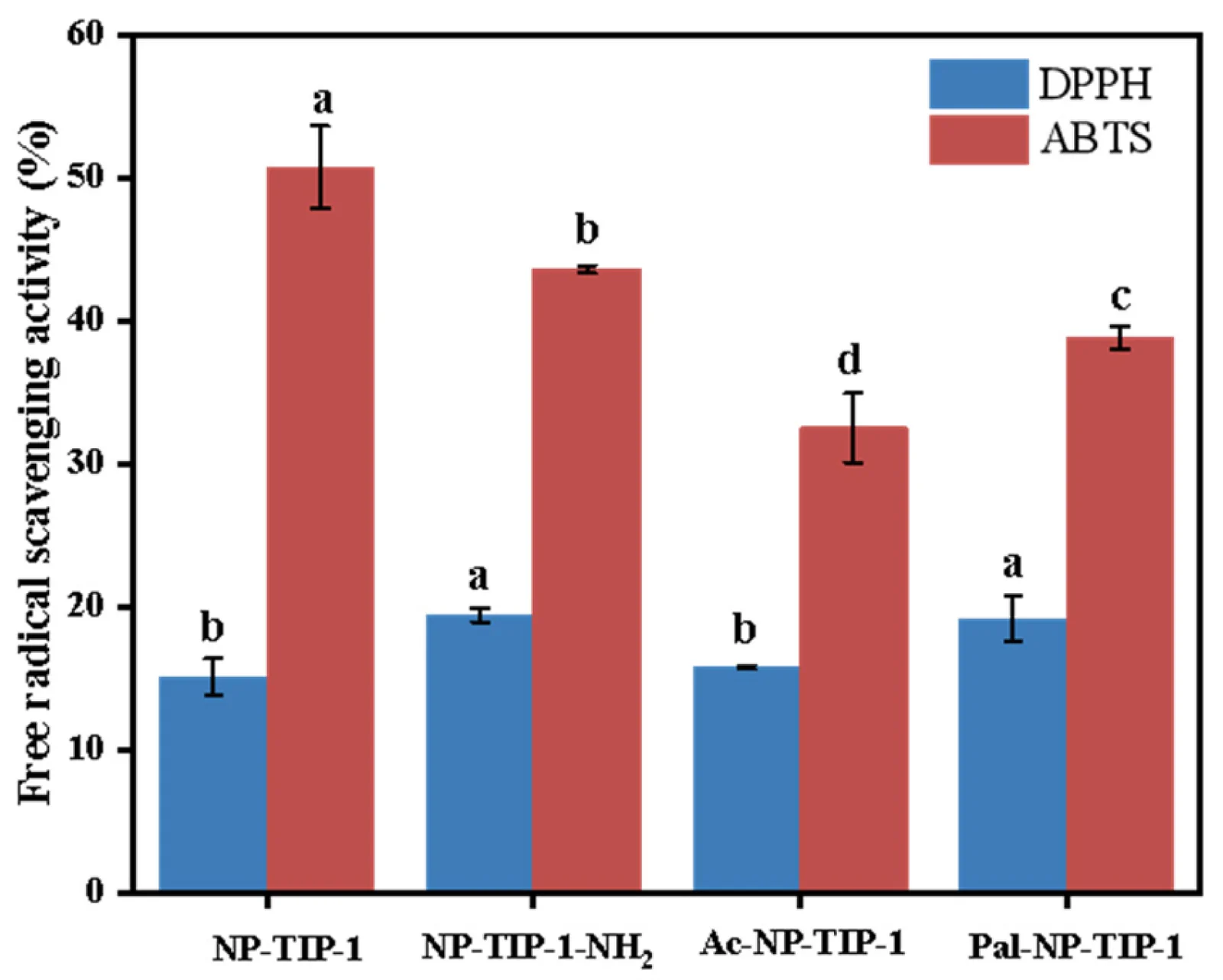
The modification of NP-TIP-1 before and after on the scavenging rates of DPPH and ABTS free radicals. Different letters indicate significant differences between data (*p* < 0.05).

As shown in Figure 1, the chemical structures of the three modified peptides are illustrated. As shown in Table 1, using kojic acid as a positive control, the synthetic peptides NP-TIP-1-NH_2_, Ac-NP-TIP-1, and Pal-NP-TIP-1 exhibited varying degrees of inhibitory activity against tyrosinase. Among these, NP-TIP-1-NH_2_ demonstrated the highest tyrosinase inhibitory activity, with an IC_50_ value of 0.979 ± 0.028 mM, which is lower than that of NP-TIP-1, indicating that the *C*-terminal amidation modification enhances the tyrosinase inhibitory activity of the NP-TIP-1. This may be because amidation modification enhances the affinity between peptides and tyrosinase [19]. The decrease in tyrosinase activity after acetylation and palmitoylation modifications may be influenced by the peptide’s own hydrophilicity, which prevents the peptide from forming a suitable reaction environment with the enzyme, thereby affecting its inhibitory effect on tyrosinase [20]. As shown in Figure 2, at a peptide concentration of 2 mg/mL, the DPPH radical scavenging capacity of NP-TIP-1-NH_2_, Ac-NP-TIP-1, and NP-TIP-1 increased from 15.39% to 19.42% and 19.15%, respectively (*p* < 0.05), while the ABTS radical scavenging ability decreased after *C*-terminal amidation, *N*-terminal acetylation, and *N*-terminal palmitoylation modifications (*p* < 0.05), from 50.76% to 43.59%, 32.51%, and 38.82%, respectively. Given that NP-TIP-1-NH_2_ is a highly hydrophilic amidated peptide, the half-maximal inhibitory concentration (IC_50_) values differed between the DPPH and ABTS assays. Overall, among the three modification strategies, C-terminal amidation was found to enhance the DPPH radical-scavenging ability of the NP-TIP-1 peptide while maintaining the optimal ABTS radical-scavenging effect. Based on the in vitro tyrosinase inhibition IC50 results, NP-TIP-1-NH_2_ was selected for subsequent experiments.

### 2.2. Thermal Stability of Amide Peptides

**Figure 3:**
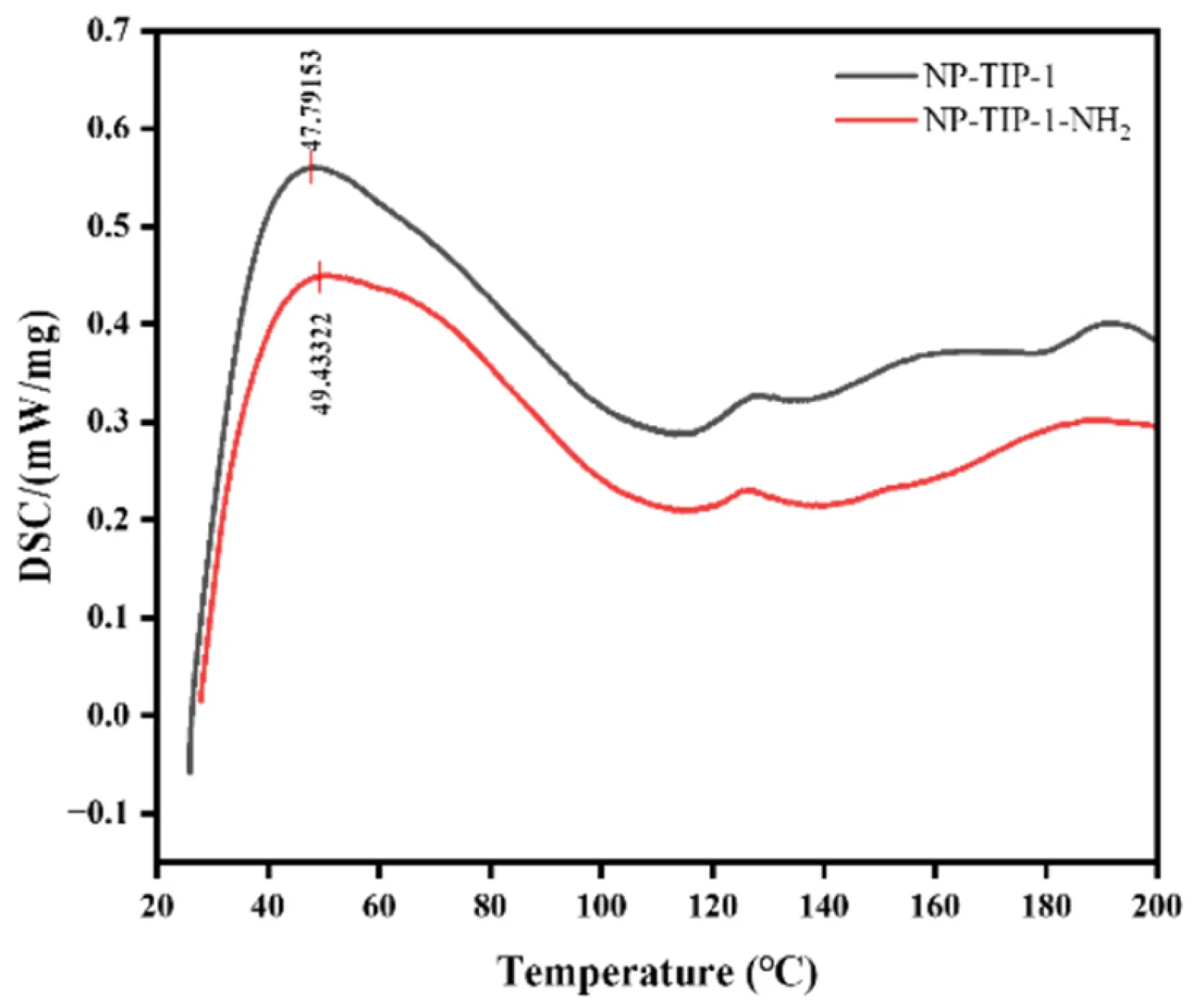
The effect of modification on the thermostability of NP-TIP-1.

TG-DSC is an analytical method that combines thermogravimetric analysis (TG) and differential scanning calorimetry (DSC) to simultaneously measure changes in sample mass and thermal effects during heating [21]. As shown in Figure 3, the decomposition temperatures of NP-TIP-1 and NP-TIP-1-NH_2_ at 20–200 °C are 47.79 °C and approximately 49.43 °C, respectively. The modified NP-TIP-1 retains its original thermal stability compared to the unmodified form, which may be attributed to the amidation modification better mimicking the original state of the carboxyl group of the parent protein in the body [22].

### 2.3. Cu^2+^ Chelation Ability of Amide Peptides

**Figure 4:**
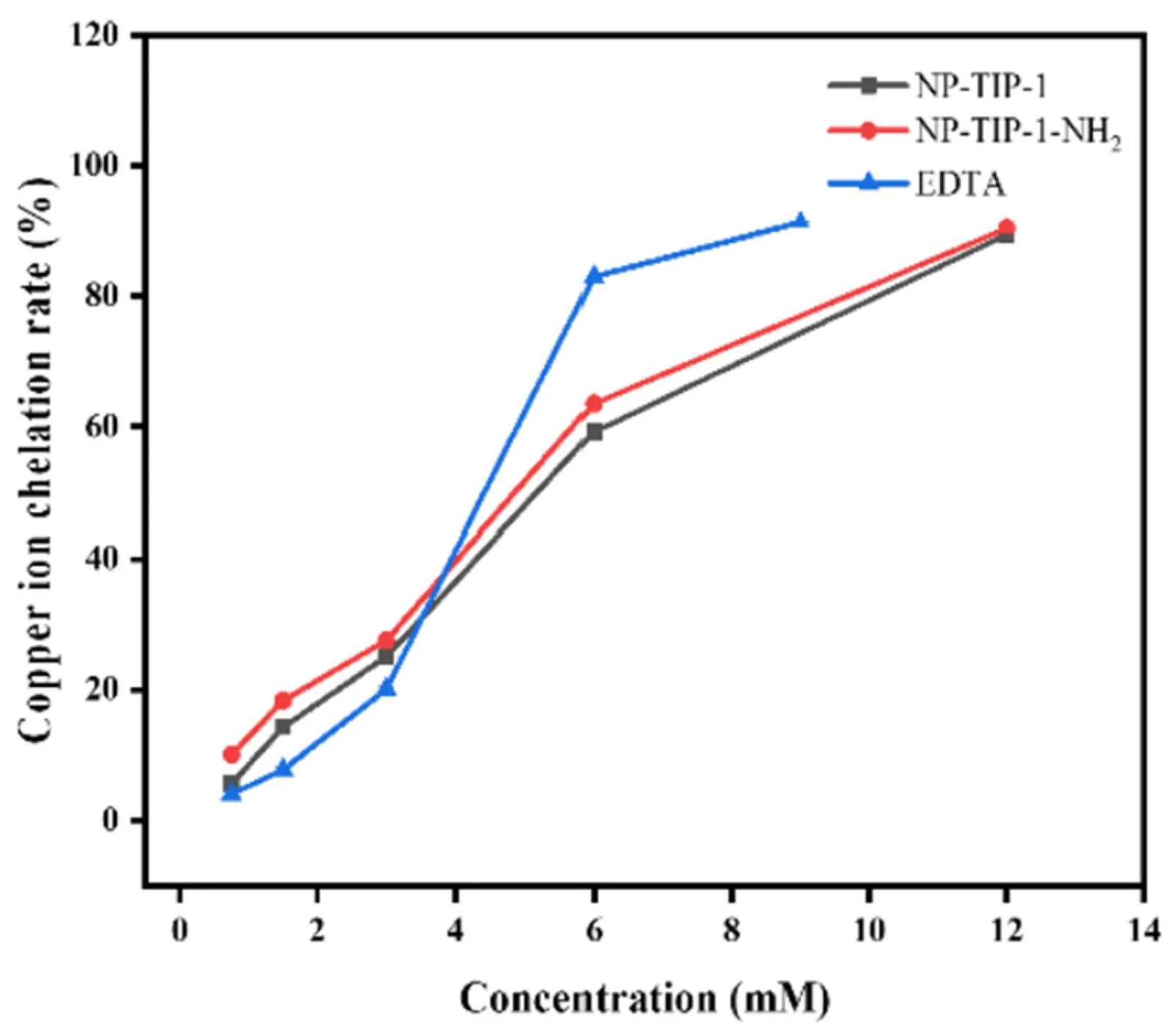
The effect of modification on the copper ion chelating ability of NP-TIP-1.

The ability of compounds to chelate the bivalent copper ions at the active site of tyrosinase can exhibit inhibitory activity against tyrosinase [23]. The chelating ability of NP-TIP-1 and NP-TIP-1-NH_2_ at different concentrations toward Cu^2+^ was determined, with the metal chelating agent EDTA serving as a positive control. The results are shown in Figure 4. As the concentration increases, the chelating ability of EDTA and NP-TIP-1 toward Cu^2+^ exhibits concentration-dependent behavior within the 0.75–12 mM concentration range. At 12 mM, the chelation rates for copper ions are 89.41% and 90.38%, respectively, and the modified compounds retain good copper ion chelating ability.

### 2.4. The Effect of NP-TIP-1-NH_2_ on Cell Viability

**Figure 5:**
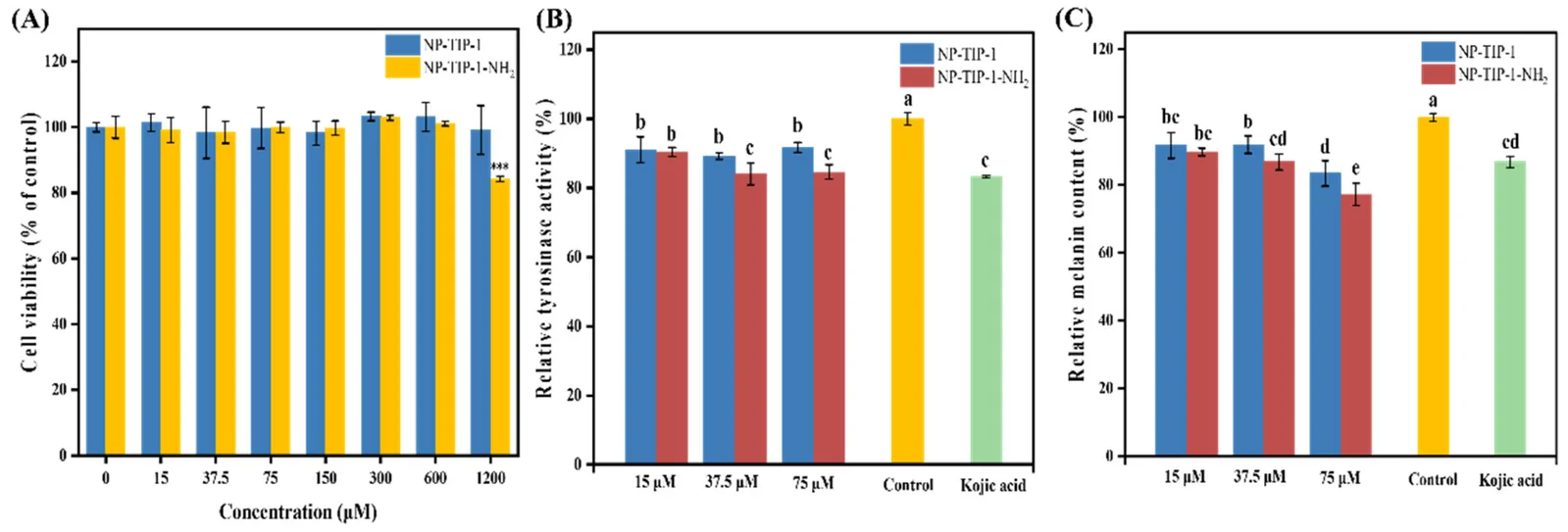
Effects of NP-TIP-1 modification on B16F10 cell activity, intracellular tyrosinase activity, and melanin content. (**A**): Effects of NP-TIP-1 modification on B16F10 cell activity; (**B**): intracellular tyrosinase activity; (**C**): melanin content. *** (*p* < 0.001) indicates a very significant difference between NP-TIP-1 and NP-TIP-1-NH_2_. Different letters indicate significant differences between data (*p* < 0.05).

Peptide modification may lead to biological toxicity, so both unmodified and amide-modified peptides were tested for their effects on B16F10 cells at certain concentrations [24]. As shown in Figure 5, within the concentration range of 15–1200 µM, the survival rate of B16F10 cells treated with NP-TIP-1 showed no significant difference compared to the control group (*p* > 0.05). NP-TIP-1-NH_2_ showed no cytotoxic effects at concentrations of 15–600 µM but had a significant impact on cell viability at 1200 µM (*p* < 0.05). Therefore, based on the above results, we selected concentrations of 15, 37.5, and 75 µM for subsequent experiments.

### 2.5. The Effect of NP-TIP-1-NH_2_ on Intracellular Tyrosinase and Melanin

**Figure 6:**
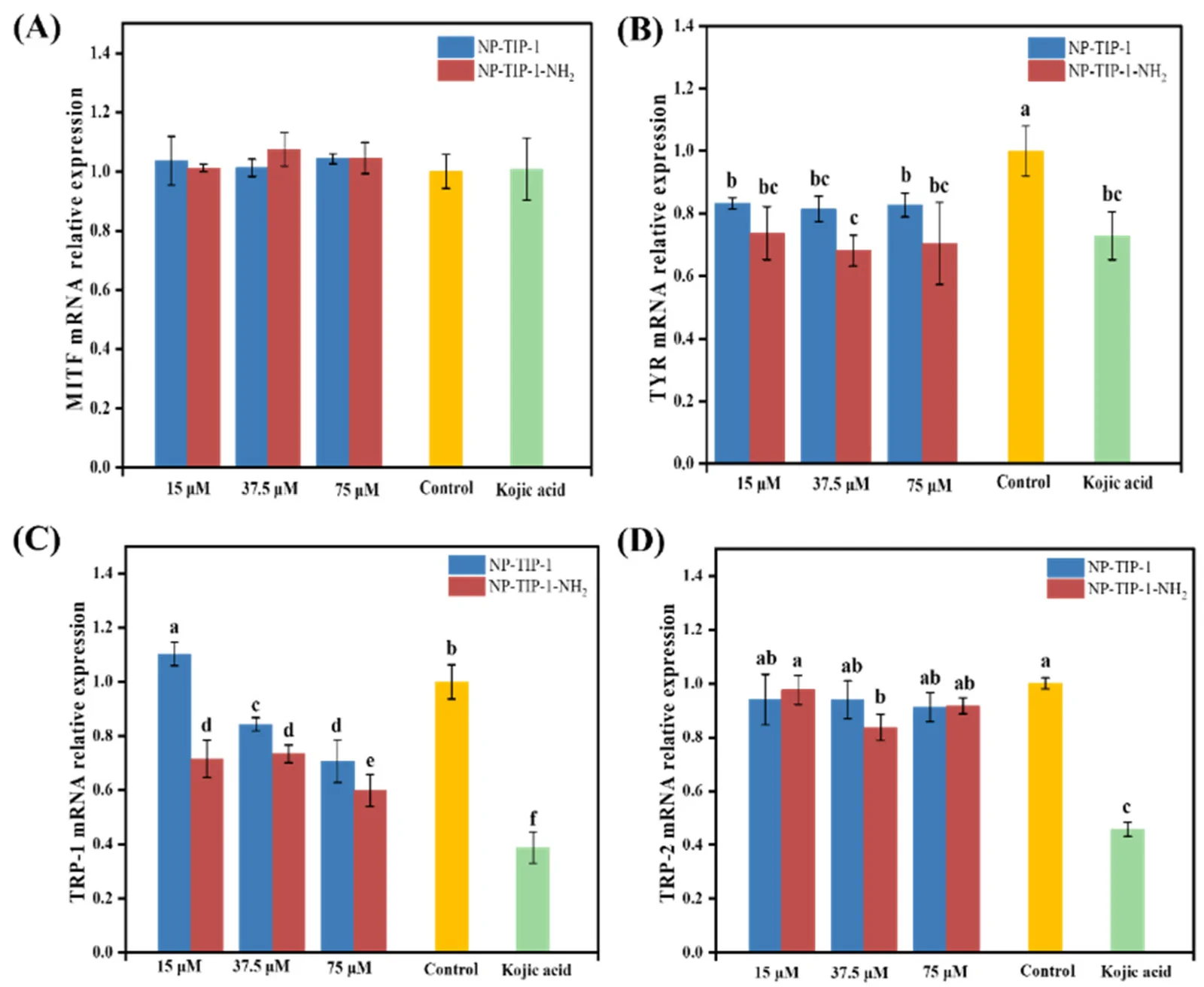
The effect of NP-TIP-1 modification on the expression of tyrosinase-related genes in B16F10 cells. (**A**): MITF; (**B**): TYR; (**C**): TRP-1; (**D**): TRP-2. Different letters indicate significant differences between data (*p* < 0.05).

As shown in Figure 6, compared to the nine-peptide or nineteen-peptide extracted from the flesh of *Pinctada martensii*, the effective concentration of the nacre peptide is significantly higher [25]. Its impact on tyrosinase activity and melanin content is concentration-dependent, with a comparable inhibitory effect. As demonstrated, short peptides may offer significant advantages in tyrosinase inhibitory activity [7]. Compared with the blank group, there were significant differences (*p* < 0.05) in tyrosinase activity and melanin content in modified cells at concentrations of 37.5 and 75 μM compared with those in unmodified cells. At concentrations of 15, 37.5, and 75 μM, compared with the blank group, enzyme activity decreased from 91.11%, 89.18%, and 91.78% to 90.4%, 84.04%, and 84.62%, respectively. The tyrosinase activity in the kojic acid treatment group was 83.34%. At 37.5 and 75 μM concentrations, the tyrosinase-inhibiting effects of the modified peptides were comparable to those of the positive control kojic acid. Melanin content in the modified groups decreased from 91.7%, 91.12%, and 83.45% to 89.78%, 86.84%, and 77.21%, respectively. The melanin content in the kojic acid treatment group was 86.84%. At a concentration of 75 μM, the effects of unmodified and amide-modified peptides on melanin content were superior to those of kojic acid.

### 2.6. The Effect of NP-TIP-1-NH_2_ on the Expression of Tyrosinase-Related Genes

To further investigate the effect of peptides on melanin production in B16F10 cells, changes in tyrosinase-related gene expression were measured. B16F10 cells were treated simultaneously with unmodified peptides and modified peptides at a concentration of 75 μM. The effects of tyrosinase inhibitory peptides NP-TIP-1 and NP-TIP-1-NH_2_ on mRNA expression are shown in Figure 6. MITF was found to be the primary regulator of melanin production, with this gene being regulated by upstream genes and controlling the expression of downstream genes (TYR, TRP1, and TRP2) [26,27]. Compared with the control group, NP-TIP-1-NH_2_ treatment downregulated the expression of TYR and TRP-1 without altering MITF levels (*p* < 0.05). At a concentration of 75 μM, TYR expression was significantly reduced by 17% in the NP-TIP-1 group, and TRP-1 expression was significantly reduced by 29.25%. In the NP-TIP-1-NH_2_ group, TYR mRNA expression was reduced by 30%, and TRP-1 mRNA expression was significantly reduced by 40.14%, which is consistent with the findings of Huang et al. on the anti-melanogenesis mechanism of peptides extracted from pearl oyster meat [12]. The lack of significant changes in MITF and TRP-2 gene expression suggests that peptides may also indirectly influence tyrosinase activity through antioxidant mechanisms, such as scavenging free radicals, enhancing antioxidant enzyme activity, and reducing malondialdehyde content, thereby ultimately affecting melanin synthesis [28].

### 2.7. The Effect of NP-TIP-1-NH_2_ on the Expression of Tyrosinase-Related Proteins

**Figure 7:**
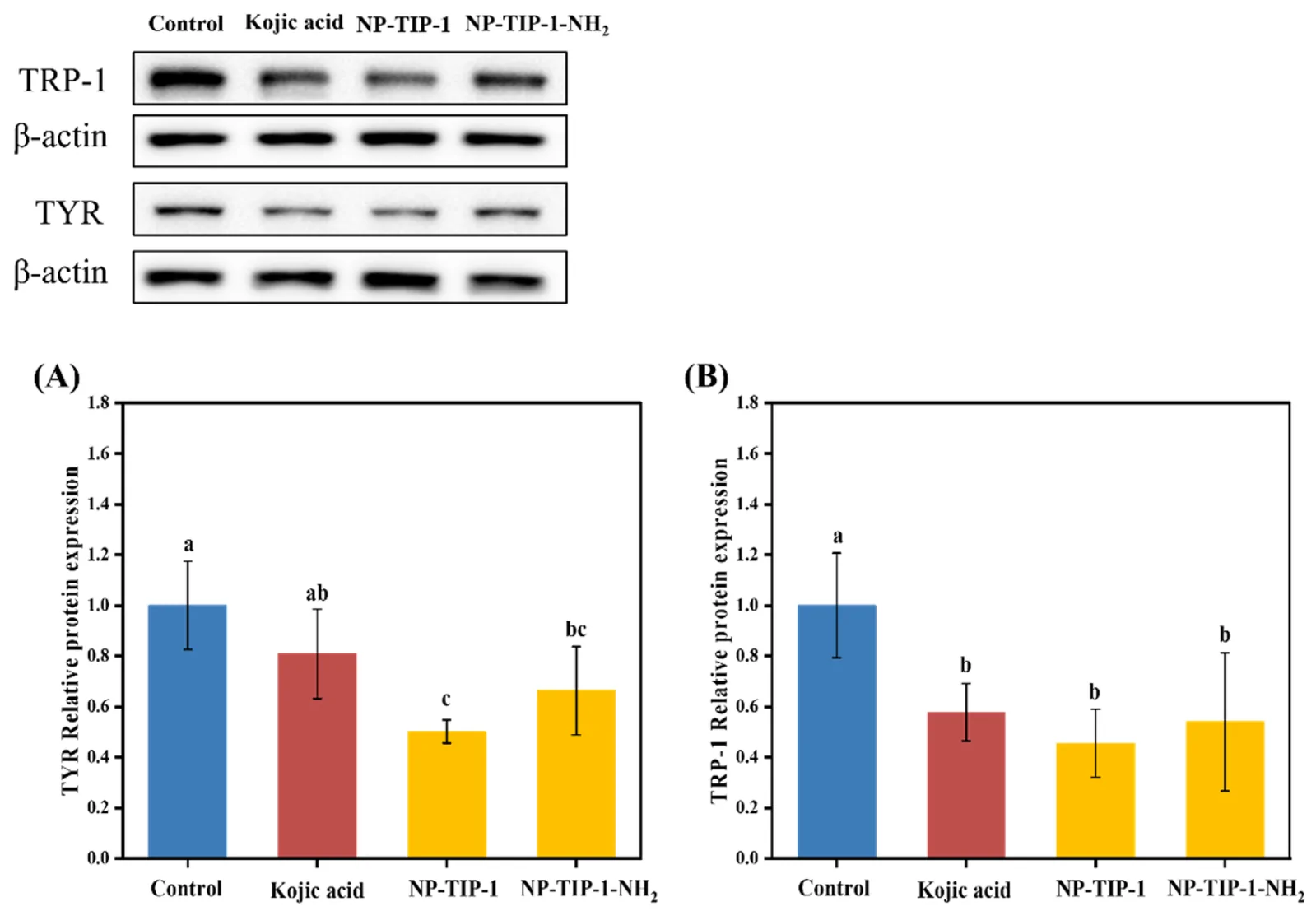
The effect of NP-TIP-1 modification on the expression of tyrosinase-related protein in B16F10 cells. (**A**): TYR; (**B**): TRP-1. Different letters indicate significant differences between data (*p* < 0.05).

To further validate the results of tyrosinase-related gene expression, B16F10 cells were simultaneously treated with unmodified peptides and modified peptides at a concentration of 75 μM. The effects of NP-TIP-1 and NP-TIP-1-NH_2_ on tyrosinase-related protein expression are shown in Figure 7, compared with the control group, both unmodified and amide-modified peptides significantly affected the expression of TYR and TRP-1 proteins in B16F10 cells (*p* < 0.05), This is similar to the findings of Chen et al. in their study on the inhibitory effects of novel tartaric acid derivatives on tyrosinase, where tartaric acid derivatives were found to significantly affect the protein expression of MITF, TYR, TRP-1, and TRP-2 in a concentration-dependent manner [29,30]. Researchers found that the effect of polyamines on melanin production indicated a reduction of approximately 70% in melanin production in B16F10 melanoma cells compared to untreated cells, but no significant effect on TYR expression, while the protein levels of TRP-1 in B16F10 cells were reduced [31]. The reduced protein expression of TRP-1 suggests that NP-TIP-1 and NP-TIP-1-NH_2_ may influence TRP-1 oxidation of 5, 6-dihydroxyindolecarboxylic acid (DHICA) to form indole-5, 6-quinone-2-carboxylic acid (ICAQ), which then polymerizes with IQ to form eumelanin. In addition, there may be antioxidant pathways that interact with tyrosinase and melanin [28,30].

### 2.8. Untargeted Metabolomics Analysis

(1) OPLS-DA analysis of the effects of pre- and post-modification on cellular metabolites compared to the blank control group

**Figure 8:**
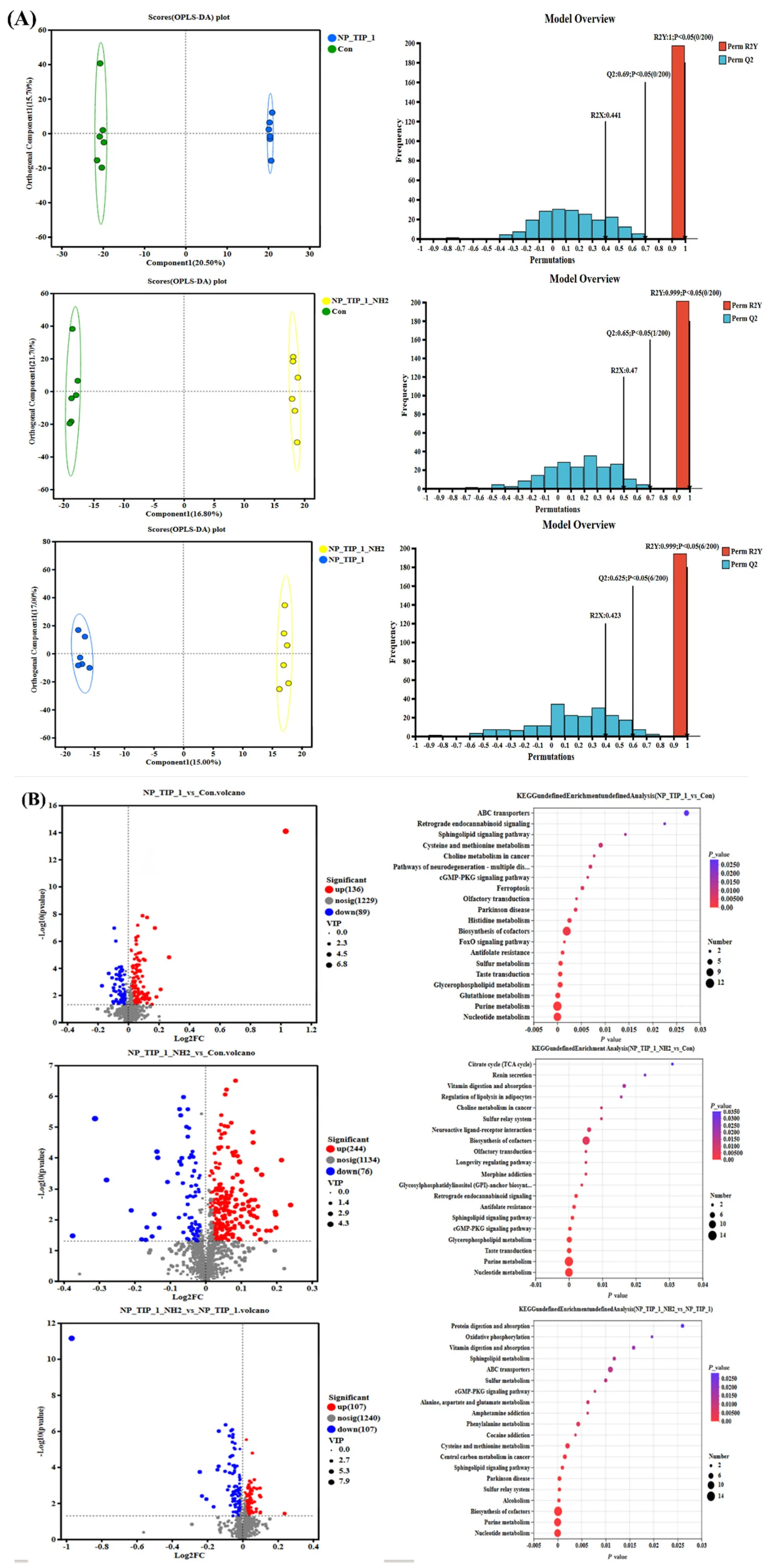

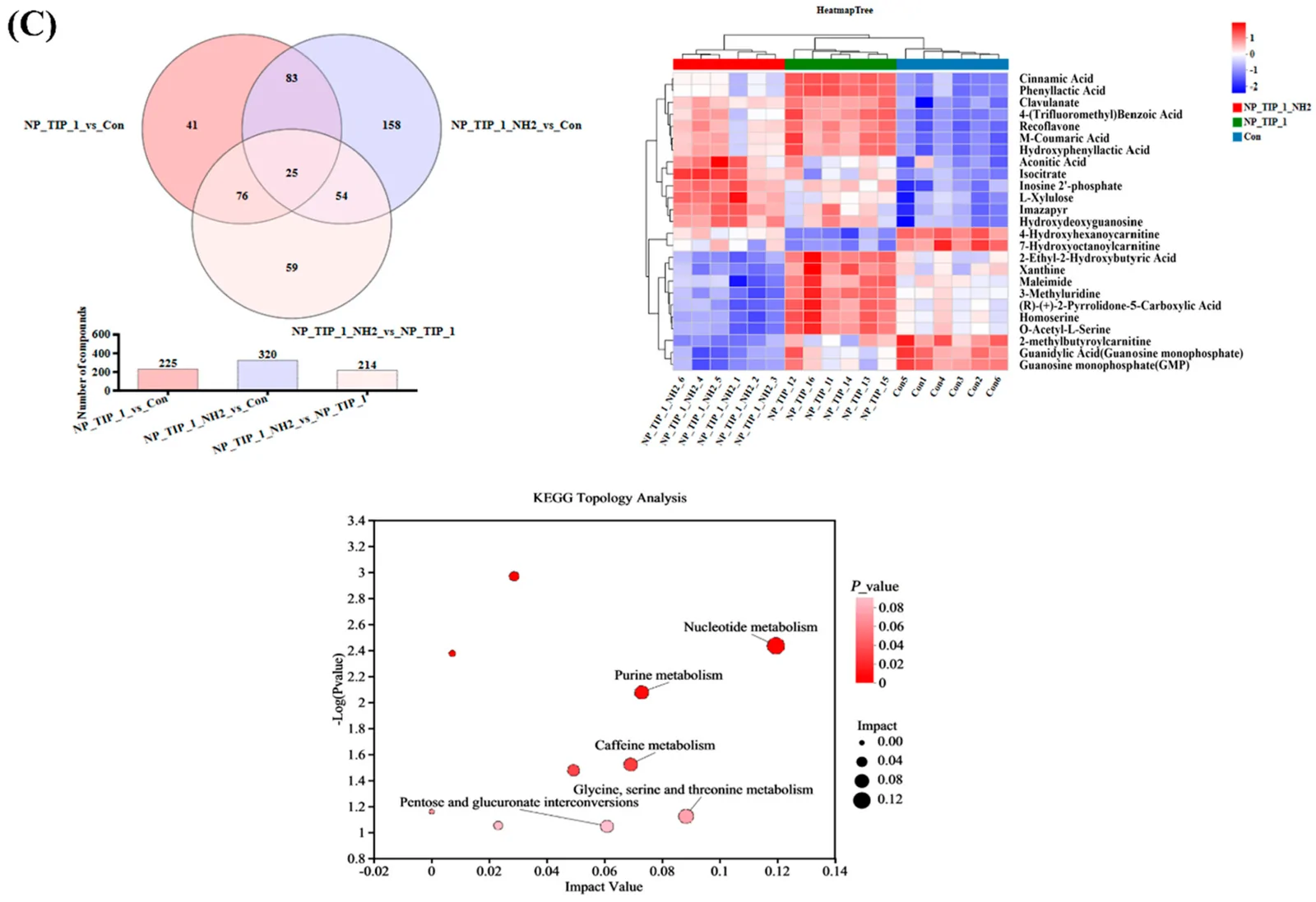
Analysis of differential metabolites; (**A**): OPLS-DA analysis with significance testing; (**B**): volcano plot and KEGG signaling pathway enrichment analysis of differential metabolites; (**C**): analysis of enriched pathways for key differential metabolites.

As shown in Figure 8A, orthogonal partial least squares discriminant analysis (OPLS-DA) was used to rigorously validate the model. The NP-TIP-1, NP-TIP-1-NH_2_, and control groups were clearly separated, and the modified group was distinctly separated from the unmodified group, with obvious clustering [32]. This indicates that there are significant differences in cellular metabolites among the groups. Replacement testing conducted 200 times supports the validity of the OPLS-DA model. The R^2^ and Q^2^ values for the three comparison groups are shown in the figure. The model parameter Q^2^ > 0.5 indicates that the established model has good predictive capability, and the modeling results are reliable [33]. Based on this, differential metabolites were screened using the criteria of VIP > 1.0 and corrected *p* < 0.05.

(2) Differential metabolite analysis

The detected differential metabolites were visualized in a volcano plot. The critical threshold for screening differential marker metabolites was a fold change > 1, VIP > 1, and *p* < 0.05. The number of differentially expressed metabolites upregulated between comparison groups ranged from 107 to 244, while the number of differentially expressed metabolites downregulated ranged from 76 to 107. As shown in Figure 8B, compared with the blank control group, the NP-TIP-1 group contained 136 up-regulated metabolites and 89 down-regulated metabolites, and the NP-TIP-1-NH_2_ group contained 244 up-regulated metabolites and 76 down-regulated metabolites, while the NP-TIP-1-NH_2_ group had 107 up-regulated metabolites and 107 down-regulated metabolites compared to the NP-TIP-1 group. Overall, compared with the blank control group, the number of differentially upregulated metabolites in the groups treated with unmodified peptides and amide-modified peptides was greater than the number of differentially downregulated metabolites, and amide modification resulted in more upregulated metabolites than unmodified peptides. This may be attributed to the fact that amide-modified peptides exert a more comprehensive influence on cells by upregulating different metabolites to multifacetedly affect metabolic pathways [34]. The metabolic pathways involved in the differentially expressed metabolites across groups are shown in Figure 8B, with the importance of metabolic pathways indicated by the ranking of enrichment significance. Among these, nucleotide metabolism, purine metabolism, and the biosynthesis pathway of cofactors are the most important. Compared to the blank group, the enriched differential metabolites in these pathways showed downregulation of cytosine (Cytosine) and guanosine monophosphate (GMP), while guanosine (Guanosine) and guanine (Guanine) were upregulated. After peptide intervention in B16F10 cells, these metabolites exhibited the same trend, suggesting they may be key metabolites. Among the top 20 metabolic pathways, the cysteine and methionine metabolic pathways, the phenylalanine metabolic pathway, and others are directly related to melanin synthesis. Additionally, glutathione metabolism is associated with melanin synthesis, primarily through regulating redox status and influencing tyrosinase activity to indirectly affect melanin production [35]. The cellular nucleotide metabolic pathway is a key process in the synthesis and degradation of nucleotides in the body, including de novo synthesis, salvage synthesis, and catabolic metabolism. These pathways not only provide raw materials for DNA and RNA synthesis but also play important roles in energy metabolism, signal transduction, and coenzyme synthesis. The increased number of differentially expressed metabolic products suggests that peptide treatment may have influenced metabolic pathways involved in the synthesis of various coenzymes and cofactors within the organism, such as regulating the antioxidant enzyme system [36]. These cofactors are essential components of many enzymes and may participate in catalyzing various biochemical reactions within B16F10 cells.

(3) Metabolic pathway enrichment analysis

Based on the Venn diagram, the differences in metabolites among the three groups are shown in the figure. Compared with the blank control group, there were 225 different metabolites in the unmodified group, 320 in the amide group, and 214 between the modified and unmodified groups, indicating that modification had a significant effect on metabolites. Among these, 25 common differentially expressed metabolites were identified as marker metabolites. The heatmap of the cluster analysis of differentially expressed metabolites is shown in Figure 8C. It can be observed that NP-TIP-1 and NP-TIP-1-NH_2_ primarily exert their effects by upregulating key differentially expressed metabolites, with the regulatory trends after modification being complementary to those before modification. Metabolic pathways were identified based on the enrichment significance and relative importance derived from topological analysis. The top three important pathways involving differential metabolites, ranked by the importance of metabolites in the pathways, are the nucleotide metabolism pathway, the glycine, serine, and threonine metabolism pathway, and the purine metabolism pathway. Researchers studied the effects of α-melanocyte-stimulating hormone (α-MSH) stimulation on B16F10 cells and found that the glycine, serine, and threonine metabolic pathways are one of the key metabolic pathways, further validating their association with melanin production [35,37]. Homoserine, xanthine, and guanosine monophosphate (GMP) are key metabolites annotated in the metabolic pathways, indicating that peptides collectively participate in nucleotide metabolism pathways, purine metabolism pathways, and others by influencing these metabolites.

The integration of metabolomic findings with molecular and cellular data reveals a coordinated multi-level anti-melanogenic mechanism of NP-TIP-1-NH_2_. The significant upregulation of guanosine and guanine, coupled with downregulation of GMP, suggests enhanced nucleotide salvage pathway activity that may redirect cellular resources from melanogenesis toward nucleotide synthesis. This metabolic shift is further supported by the perturbation of glycine/serine/threonine metabolism, as these amino acids serve as precursors for both purine biosynthesis and glutathione synthesis. The resulting increase in glutathione availability would enhance cellular antioxidant capacity, maintaining redox homeostasis. Notably, the nucleotide metabolism pathway may intersect with tyrosinase regulation through shared copper ion utilization—elevated nucleotide synthesis demands could potentially affect copper availability for tyrosinase activation, which might synergize with the direct copper chelation mechanism identified in our biophysical studies. However, this hypothesis requires further experimental validation. Furthermore, the downregulation of cytosine implies potential modulation of cytidine triphosphate (CTP) pools, which could affect phospholipid biosynthesis and membrane integrity in melanosomes, thereby impairing melanin deposition. These metabolic alterations collectively suggest that NP-TIP-1-NH_2_ may have the potential to function as a metabolic reprogramming agent that targets melanogenesis through both direct enzymatic inhibition and indirect metabolic pathway modulation.

### 2.9. Statistical Analysis

All data are expressed as the mean ± standard deviation (SD) of at least three different experiments. Multiple group comparisons were conducted using one-way analysis of variance (ANOVA) and Duncan’s multiple-range test in IBM SPSS Statistics 27. A *p*-value less than 0.05 was considered statistically significant. The graphical abstracts were created using Figdraw 2.0.

## 3. Materials and Methods

### 3.1. Chemical Modification of Peptides

The synthesis and chemical modification of peptides were carried out with the assistance of Shanghai Amlink Biotechnology Co., Ltd. (Shanghai, China). Following synthesis, the following peptides were obtained: C-terminally amide-modified peptide NP-TIP-1-NH_2_, *N*-terminally acetylated peptide Ac-NP-TIP-1, and *N*-terminally palmitoylated peptide Pal-NP-TIP-1. Purity was verified using RP-HPLC coupled with ESI/MS mass spectrometry purchased from Thermo Fisher Scientific (Waltham, MA, USA), with purity > 98%.

### 3.2. In Vitro Tyrosinase Inhibitory Activity and Antioxidant Activity

Tyrosinase (from mushroom, 500 U/mg) and PBS buffer were purchased from Shanghai Yuanye Biotechnology Co., Ltd. (Shanghai, China). L-tyrosine was obtained from Shanghai Macklin Biochemical Technology Co., Ltd. (Shanghai, China). The determination method is consistent with that described in the reference [13]. A solution of L-tyrosine (0.5 mg/mL), the sample solution, and PBS buffer were added sequentially to a 96-well plate, thoroughly mixed, and incubated at 37 °C for 10 min. Subsequently, 20 µL of tyrosinase solution (500 U/mL) was added to each well, and the reaction was conducted at 37 °C for 10 min ± 5 s. The plate was then immediately placed in an enzyme-linked immunosorbent assay (ELISA) reader purchased from Thermo Fisher Scientific (Waltham, MA, USA), and the optical density (OD) value was measured at 475 nm.

Configure NP-TIP-1 and three modified peptide concentrations at 2 mg/mL. Assess the DPPH free radical scavenging ability and the ABTS free radical scavenging ability, following the instructions provided in the Grace Biotechnology Co., Ltd. (Suzhou, China) for the testing method.

### 3.3. Thermal Stability of NP-TIP-1-NH_2_

To determine whether unstable phenomena occur before and after peptide modification, a thermogravimetric analyzer purchased from NETZSCH-Gerätebau GmbH(Selb, Germany) was employed to assess the thermal stability of the peptide both prior to and following modification [38]. Weigh an appropriate amount of the sample into an aluminum oxide cup. Set the thermal scanning range to 30–200 °C, with nitrogen (N_2_) as the purge gas at a flow rate of 20 mL/min and a heating rate of 5 °C/min.

### 3.4. Determination of Cu^2+^ Chelating Ability of NP-TIP-1-NH_2_

Determining the copper ions’ chelating ability is crucial for the development of tyrosinase inhibitors [39]. A 0.5 mL aliquot of a 1 mg/mL sample solution was mixed with 1.4 mL of 0.05 mol/L sodium acetate buffer (pH 6.0), 0.03 mL of 4 mmol/L phenol red, and 0.05 mL of 1 mg/mL CuSO_4_·5H_2_O, and the mixture was thoroughly mixed. Distilled water served as the blank, and the absorbance was measured at 632 nm. The copper ion chelation rate of the sample was calculated using the appropriate formula.

### 3.5. Cytotoxicity of NP-TIP-1-NH_2_ on B16F10 Cells

B16F10 cells used in this study was purchased from FengHui Biotechnology Co., Ltd., (Hunan, China; catalog number: CL0039).The determination method is consistent with that described in the reference [13]. B16F10 cells between passages 10 and 25 were cultured at 37 °C in a humidified 5% CO_2_ incubator in complete culture medium (DMEM/F-12 supplemented with 10% fetal bovine serum and 1% penicillin-streptomycin solution). Cell viability was assessed using the CCK-8 assay kit purchased from Zeta Life (Menlo Park, CA, USA). During the logarithmic growth phase, 100 µL of cell suspension was distributed into a 96-well plate at a density of 3000 cells per well. After incubating at 37 °C for 24 h, the supernatant was removed, and 100 µL of complete medium containing varying concentrations of NP-TIP-1 and NP-TIP-1-NH_2_ was added. A blank control was included with 100 µL of complete medium. Cell viability was measured after 48 h of treatment.

### 3.6. The Effect of NP-TIP-1-NH_2_ on Tyrosinase Activity in B16F10 Cells

The determination method is consistent with that described in the reference [13]. Cell culture conditions remain consistent with the previously described methods. Utilize a 6-well plate for cell seeding, maintaining a cell density of 2 × 10^5^ cells per well. After incubating at 37 °C for 24 h, remove the supernatant and add 2 mL of complete medium containing samples with varying concentrations of NP-TIP-1 and NP-TIP-1-NH_2_. For the blank control, add 2 mL of complete medium without any samples. The positive control group was kojic acid. After an additional 48 h, lyse the cells and wash them 1–2 times with pre-chilled PBS. Add 400–600 µL of lysis buffer (RIPA lysis buffer with PMSF at a ratio of 100:1), and collect the cells using a cell scraper into a 1.5 mL centrifuge tube. Shake the tube on ice for 30 min, then centrifuge at 4 °C at 12,000 rpm for 10 min to collect the supernatant. Standardize the protein concentration using the BCA assay kit purchased from Beyotime Biotechnology Co., Ltd. (Shanghai, China). Prepare a 1 mg/mL L-dopa solution to serve as the substrate. In a 96-well plate, add 50 µL of PBS, 50 µL of the L-dopa solution, and 50 µL of the cell lysate supernatant. Incubate the mixture at 37 °C for 1 h, then measure dopamine formation at 475 nm. Tyrosinase activity is expressed as a percentage relative to the blank control.

### 3.7. The Effect of Amide Peptides on Melanin Content in B16F10 Cells

The determination method is consistent with that described in the reference [13]. Cell culture conditions were consistent with those described previously. Cells were seeded in 6-well plates at a density of 2 × 10^5^ cells per well. After incubation at 37 °C for 24 h, the supernatant was removed, and 2 mL of complete medium containing varying concentrations of NP-TIP-1 and NP-TIP-1-NH_2_ samples was added. For the blank control, 2 mL of complete medium was added. The positive control group was kojic acid (50 µg/mL). Following 48 h of treatment, the cells were digested with trypsin, and the cell pellet was collected. A 1 mol/L sodium hydroxide solution containing 10% DMSO was added to the cell pellet, and the mixture was reacted at 80 °C in a water bath for 1 h. The cell lysate was then centrifuged at 12,000 rpm for 10 min. A total of 200 µL of the supernatant was transferred to a 96-well plate, and the absorbance was measured at 405 nm. Melanin content is expressed as a percentage of the control group.

### 3.8. Tyrosinase-Related Gene Expression in B16F10 Cells

Following the method described by Huang et al. with minor modifications [12], cells were seeded in 6-well plates at a density of 2 × 10^5^ cells per well. After incubation at 37 °C for 24 h, the supernatant was removed, and 2 mL of complete medium containing various concentrations of NP-TIP-1 and NP-TIP-1-NH_2_ samples was added. A blank control was included, consisting of 2 mL of complete medium. After 48 h of treatment, total RNA was extracted from B16F10 cells using the FreeZol Reagent lysis method. The concentration and purity of the total RNA were assessed using a micro-spectrophotometer Purchased from Beijing Kaiao Technology Development Co., Ltd. (Beijing, China) to confirm RNA integrity. The expression levels of relevant genes, including MITF (Microphthalmia-associated Transcription Factor), TYR, TRP1, TRP2, and β-actin, were measured using quantitative fluorescent PCR Purchased from Bio-Rad Life Medical Products Co., Ltd. (Shanghai, China). The PCR amplification program was pre-set at 95 °C, followed by denaturation at 95 °C for 30 s, annealing at 60 °C for 30 s, and extension at 72 °C for 30 s, repeated for 40 cycles. The primer sequences are as follows: β-actin (5′-CTGGTCGTACCACAGGCATT-3′, 5′-ATGTCACGCACGATTTCCCT-3′), MITF (5′-ACAGCAACGAGCTAAGGACC-3′, 5′-CTCTAGCCTGCATCTCCAGC-3′), TYR (5′-TCCTTCTTCTCCTCCTGGCA-3′, 5′-TGGGGGTTTTGGCTTTGTCA-3′), TRP-1 (5′-TTCATTGGCACCTGCTTTGC-3′, 5′-TCACAGCTCCAACGAAGGAC-3′), TRP-2 (5′-TGGTGGACATGCCTCAGTTC-3′, 5′-CAGGTAGAGCTTGGAGCGAG-3′). β-actin was used as the internal reference gene, and the 2-ΔΔCT method was employed to calculate the expression of target genes.

### 3.9. Expression of Tyrosinase-Related Protein in B16F10 Cells

Following the method established by Han et al. [40], with slight modifications, protein immunoblotting analysis was conducted (Image J 2fiji 1.54p). Cells were treated with tyrosinase inhibitory peptides NP-TIP-1 and NP-TIP-1-NH_2_ (75 μM), alongside a peptide-free control group and a positive control group (kojic acid). Cells were lysed using RIPA lysis buffer, and protein concentration was determined using the BCA protein assay kit. After preparing the gel, 25 µg of protein was subjected to SDS-PAGE electrophoresis, which was performed at a constant voltage of 90 V until the bromophenol blue band reached the bottom of the gel. PVDF membranes were pretreated to facilitate the gel transfer process, which was executed at a constant current of 300 mA and at a low temperature. The membranes were blocked for 1 h and subsequently washed for 40 min. The bands were incubated with the primary antibody overnight at 4 °C, followed by incubation with the secondary antibody for 1 h at 37 °C. Finally, the membranes were washed for an additional 40 min, and the bands were developed.

### 3.10. Non-Targeted Metabolomics

Select B16F10 cells from the 10th to 25th passages and culture them in a 5% CO_2_ incubator at 37 °C using a culture medium composed of DMEM/F-12 supplemented with 10% fetal bovine serum and 1% penicillin-streptomycin solution. During the logarithmic growth phase, distribute 8 mL of cell suspension into 10 cm culture dishes, ensuring a density of 5 × 10^5^ cells per well. After incubating at 37 °C for 24 h, remove the supernatant and add 8 mL of complete medium containing varying concentrations of NP-TIP-1 and NP-TIP-1-NH_2_. The blank control should receive 100 µL of complete medium. Collect the cells after 48 h of treatment. Non-targeted metabolomics analysis will be conducted by Shanghai Meiji Biotechnology Co., Ltd. (Shanghai, China).

(1) Select B16F10 cells from the 10th to 25th passages and culture them in a 5% CO_2_ incubator at 37 °C using a culture medium composed of DMEM/F-12 supplemented with 10% fetal bovine serum and 1% penicillin-streptomycin solution. During the logarithmic growth phase, distribute 8 mL of the cell suspension into 10 cm culture dishes, ensuring a density of 5 × 10^5^ cells per dish. After incubating at 37 °C for 24 h, remove the supernatant and add 8 mL of complete medium containing varying concentrations of NP-TIP-1 and NP-TIP-1-NH_2_. The blank control should receive 100 µL of complete medium. Collect the cells after 48 h of treatment. Non-targeted metabolomics analysis will be conducted by Shanghai Meiji Biotechnology Co., Ltd.
(2) LC-MS Detection: The LC-MS analysis was conducted using a UHPLC QExactive HF-X system (Thermo Fisher Scientific Inc., Guangzhou, China). The chromatographic conditions are as follows: the chromatographic column used was the ACQUITY UPLCHSST3 (100 mm × 2.1 mm i.d., 1.8 µm; Waters, Milford, MA, USA). The mobile phase A consisted of 95% water and 5% acetonitrile (with 0.1% formic acid), while mobile phase B comprised 47.5% acetonitrile, 47.5% isopropanol, and 5% water (also containing 0.1% formic acid). The injection volume was 3 μL, and the column temperature was maintained at 40 °C. For mass spectrometry conditions, samples were ionized using electrospray ionization, and mass spectrometry signals were collected in both positive and negative ion scanning modes. The scanning range was set from 70 to 1050 m/z, with the ion spray voltage adjusted to 3500 V for both positive and negative ions. The sheath gas flow rate was set to 50 arb, and the auxiliary gas flow rate was 13 arb. The ion source was heated to 425 °C, the capillary temperature was maintained at 325 °C, and the collision energy was set to 20%, 40%, and 60%. The resolution was 60,000, and the MS2 resolution was 7500.
(3) Data Preprocessing: Raw data were imported into the metabolomics processing software Progenesis QI v3.0 (Waters Corporation, Milford, MA, USA) for baseline filtering, peak identification, integration, retention time correction, peak alignment, and other operations. This process ultimately yielded a data matrix containing information such as retention time, mass-to-charge ratio, and peak intensity. Subsequently, the software was utilized for feature peak library identification, matching MS and MS/MS mass spectrometry data with metabolic databases. The mass error for MS was set to less than 10 ppm, and metabolites were identified based on secondary mass spectrometry matching scores. The primary databases included well-known public resources such as http://www.hmdb.ca/, https://metlin.scripps.edu/, and a custom-built database (accessed on 24 December 2024).
(4) Data Analysis: Principal Component Analysis (PCA) and Orthogonal Partial Least Squares Discriminant Analysis (OPLS-DA) were conducted on the preprocessed data matrix utilizing the ropls package (Version 1.6.2) in R. The stability of the model was evaluated through 7-fold cross-validation. Significantly different metabolites were identified based on the Variable Importance in Projection (VIP) values derived from the OPLS-DA model and the *p*-values obtained from Student’s t-test. Metabolites with VIP > 1 and *p* < 0.05 were classified as significantly different. Differential metabolites were annotated for metabolic pathways using the KEGG database (https://www.kegg.jp/kegg/pathway.html) (accessed on 24 December 2024). to elucidate the pathways associated with these metabolites. Pathway enrichment analysis was performed using the Python3.10 package scipy.stats, and the biological pathways most pertinent to the experimental treatment were identified through Fisher’s exact test.

## 4. Conclusions

This study successfully enhanced the tyrosinase inhibitory potency of nacre-derived peptide NP-TIP-1 through *C*-terminal amidation, achieving a 2-fold improvement in IC50 while preserving its physicochemical stability and safety profile. The structure-activity relationship analysis revealed that the terminal modification site critically determines inhibitory efficacy, with *C*-terminal amidation outperforming *N*-terminal alternatives. Mechanistic investigations in B16F10 cells demonstrated that NP-TIP-1-NH_2_ exerts anti-melanogenic effects through an integrated triple-mode mechanism: (i) downregulating the expression of TYR and TRP-1 at both the transcriptional and translational levels without affecting MITF, suggesting a potential regulatory mechanism independent of MITF-mediated transcriptional control; (ii) metabolic reprogramming characterized by significant perturbation of nucleotide metabolism (upregulation of guanosine/guanine and downregulation of GMP/cytosine), glycine/serine/threonine metabolism, and purine metabolism, which collectively redirect cellular metabolic flux away from melanogenesis while enhancing antioxidant capacity through glutathione precursor availability; and (iii) potential competitive copper sequestration via enhanced nucleotide biosynthesis demands, synergizing with the intrinsic copper chelation capacity of the peptide. The bidirectional regulation of these interconnected pathways—enzymatic, transcriptional-translational, and metabolic—establishes NP-TIP-1-NH_2_ as a multifunctional modulator rather than a simple enzyme inhibitor. Future studies should first investigate the upstream signaling mechanisms underlying NP-TIP-1-NH_2_-mediated regulation of TYR and TRP-1 expression by detecting the phosphorylation levels of key signaling proteins (e.g., ERK, AKT, CREB, β-catenin, and cAMP) using Western blotting. The inhibitory activity of NP-TIP-1-NH_2_ against tyrosinase should also be further validated in B16F10 cell lysates and human primary melanocytes, and its efficacy should be evaluated in 3D skin models or animal models. Furthermore, in vivo studies employing animal models combined with isotope-tracing metabolomics are warranted to confirm these mechanisms and quantify metabolic flux changes, thereby facilitating the translational application of NP-TIP-1-NH_2_ in functional foods and cosmeceutical products.

## Figures and Tables

**Table 1:** Modified peptides inhibit the activity of tyrosinase monophenolase.

	Peptide	IC50 (mM)
unmodified peptide	NP-TIP-1	2.012 ± 0.088 b
Modified peptide	NP-TIP-1-NH_2_	0.979 ± 0.028 c
	Ac-NP-TIP-1	3.430 ± 0.017 a
	Pal-NP-TIP-1	—
Kojic acid		0.01 ± 0.003 d

Note: Different letters indicate significant differences between data (*p* < 0.05).

## Data Availability

The original contributions presented in this study are included in the article. Further inquiries can be directed to the corresponding author.

## References

[1] Cordero R.J.B., Casadevall A. (2020). Melanin. Curr. Biol..

[2] Schadendorf D., van Akkooi A.C.J., Berking C., Griewank K.G., Gutzmer R., Hauschild A., Ugurel S. (2018). Melanoma. Lancet.

[3] Hida T., Kamiya T., Kawakami A., Ogino J., Sohma H., Uhara H., Jimbow K. (2020). Elucidation of Melanogenesis Cascade for Identifying Pathophysiology and Therapeutic Approach of Pigmentary Disorders and Melanoma. Int. J. Mol. Sci..

[4] Wang Y., Xiong B., Xing S., Chen Y., Liao Q., Mo J., Sun H. (2023). Medicinal Prospects of Targeting Tyrosinase: A Feature Review. Curr. Med. Chem..

[5] Wakamatsu K., Zippin J.H., Ito S. (2021). Chemical and biochemical control of skin pigmentation with special emphasis on mixed melanogenesis. Pigment. Cell Melanoma Res..

[6] Nakajima H., Nagata T., Koga S., Imokawa G. (2014). Reduced glutathione disrupts the intracellular trafficking of tyrosinase and tyrosinase-related protein-1 but not dopachrome tautomerase and Pmel17 to melanosomes, which results in the attenuation of melanization. Arch. Dermatol. Res..

[7] Song Y., Chen S., Li L., Zeng Y., Hu X. (2022). The Hypopigmentation Mechanism of Tyrosinase Inhibitory Peptides Derived from Food Proteins: An Overview. Molecules.

[8] Tung X.Y., Yip J.Q., Gew L.T. (2023). Searching for Natural Plants with Antimelanogenesis and AntityrosinaseProperties for Cosmeceutical or Nutricosmetics Applications: A Systematic Review. ACS Omega.

[9] Wang J., Wang Y., Tang Q., Wang Y., Chang Y., Zhao Q., Xue C. (2010). Antioxidation activities of low-molecular-weight gelatin hydrolysate isolated from the sea cucumber *Stichopus japonicus*. J. Ocean Univ. China.

[10] Hu Z.-Z., Ma T.-X., Sha X.-M., Zhang L., Tu Z.-C. (2022). Improving tyrosinase inhibitory activity of grass carp fish scale gelatin hydrolysate by gastrointestinal digestion: Purification, identification and action mechanism. LWT—Food Sci. Technol..

[11] Thaha A., Wang B.-S., Chang Y.-W., Hsia S.-M., Huang T.-C., Shiau C.-Y., Chen T.-Y. (2021). Food-Derived Bioactive Peptides with Antioxidative Capacity, Xanthine Oxidase and Tyrosinase Inhibitory Activity. Processes.

[12] Huang P., Miao J., Liao W., Huang C., Chen B., Li Y., Cao Y. (2023). Rapid screening of novel tyrosinase inhibitory peptides from a pearl shell meat hydrolysate by molecular docking and the anti-melanin mechanism. Food Funct..

[13] Li F., Lin H., Qin X., Gao J., Chen Z., Cao W., Xie S. (2024). In Silico Identification and Molecular Mechanism of Novel Tyrosinase Inhibitory Peptides Derived from Nacre of *Pinctada martensii*. Mar. Drugs.

[14] Bhunia S., Purushotham M., Karan G., Paul B., Maji M.S. (2023). Exploring Chemical Modifications of Aromatic Amino Acid Residues in Peptides. Synthesis.

[15] Bednarska N.G., Wren B.W., Willcocks S.J. (2017). The importance of the glycosylation of antimicrobial peptides: Natural and synthetic approaches. Drug Discov. Today.

[16] Koller T.O., Morici M., Berger M., Safdari H.A., Lele D.S., Beckert B., Wilson D.N. (2023). Structural basis for translation inhibition by the glycosylated drosocin peptide. Nat. Chem. Biol..

[17] Cui Z., Yu Y., Zhou T., Qi C., Gu J., Zhang N., Liu Y. (2025). Cyclization: A potential effective modification strategy for umami peptides. Food Chem..

[18] Sharma R., Tomar S., Puri S., Wangoo N. (2022). Self-Assembled Peptide Hydrogel for Accelerated Wound Healing: Impact of *N*-Terminal and *C*-Terminal Modifications. Chembiochem.

[19] Okamoto T., Schlegel A., Scherer P.E., Lisanti M.P. (1998). Caveolins, a family of scaffolding proteins for organizing “preassembled signaling complexes” at the plasma membrane. J. Biol. Chem..

[20] Cao R., Li L., Xu Z., Li J., Wu D., Wang Y., Zhu H. (2023). The lipidation and glycosylation enabling bioactivity enhancement and structural change of antibacterial peptide G3. Bioorganic Med. Chem. Lett..

[21] Zhao L., Huang Q., Huang S., Lin J., Wang S., Huang Y., Rao P. (2014). Novel Peptide with a Specific Calcium-Binding Capacity from Whey Protein Hydrolysate and the Possible Chelating Mode. J. Agric. Food Chem..

[22] Eipper B.A., Stoffers D.A., Mains R.E. (1992). The biosynthesis of neuropeptides: Peptide alpha-amidation. Annu. Rev. Neurosci..

[23] Akyilmaz E., Yorganci E., Asav E. (2010). Do copper ions activate tyrosinase enzyme? A biosensor model for the solution. Bioelectrochemistry.

[24] Rana M., Maurya P., Reddy S.S., Singh V., Ahmad H., Dwivedi A.K., Barthwal M.K. (2016). A Standardized Chemically Modified *Curcuma longa* Extract Modulates IRAK-MAPK Signaling in Inflammation and Potentiates Cytotoxicity. Front. Pharmacol..

[25] Meng J., Liu J., Lu J., Jiang P., Bai Y., Liu X., Li S. (2023). Isolation, identification, and preparation of tyrosinase inhibitory peptides from *Pinctada martensii* meat. Biotechnol. Lett..

[26] Lee M.-K., Moon K.M., Park S.-Y., Seo J., Kim A.-R., Lee B. (2024). 10(E)-Pentadecenoic Acid Inhibits Melanogenesis Partly Through Suppressing the Intracellular MITF/Tyrosinase Axis. Antioxidants.

[27] Wan P., Hu Y., He L. (2011). Regulation of melanocyte pivotal transcription factor MITF by some other transcription factors. Mol. Cell. Biochem..

[28] Hu Z., Sha X., Zhang L., Huang S., Tu Z. (2022). Effect of Grass Carp Scale Collagen Peptide FTGML on cAMP-PI3K/Akt and MAPK Signaling Pathways in B16F10 Melanoma Cells and Correlation between Anti-Melanin and Antioxidant Properties. Foods.

[29] Chen Y.-M., Su W.-C., Li C., Shi Y., Chen Q.-X., Zheng J., Wang Q. (2019). Anti-melanogenesis of novel kojic acid derivatives in B16F10 cells and zebrafish. Int. J. Biol. Macromol..

[30] Nishioka E., Funasaka Y., Kondoh H., Chakraborty A.K., Mishima Y., Ichihashi M. (1999). Expression of tyrosinase, TRP-1 and TRP-2 in ultraviolet-irradiated human melanomas and melanocytes: TRP-2 protects melanoma cells from ultraviolet B induced apoptosis. Melanoma Res..

[31] Verdoni M., Roudaut H., De Pomyers H., Gigmes D., Bertin D., Luis J., Mabrouk K. (2016). ArgTX-636, a polyamine isolated from spider venom: A novel class of melanogenesis inhibitors. Bioorganic Med. Chem..

[32] Yu Z., Huang M., Clowers B.H. (2018). Comparative metabolite profiling of a metastatic and primary melanoma cell line using untargeted metabolomics: A case study. Clin. Mass Spectrom..

[33] Pena-Martin J., Garcia-Ortega M.B., Palacios-Ferrer J.L., Diaz C., Garcia Ma Boulaiz H., Marchal J.A. (2024). Identification of novel biomarkers in the early diagnosis of malignant melanoma by untargeted liquid chromatography coupled to high-resolution mass spectrometry-based metabolomics: A pilot study. Br. J. Dermatol..

[34] Zhao H., Chen H., Wu G., Xu J., Zhu W., Chen J., Guo S. (2025). Integrative metabolomics-GC-IMS approach to assess metabolic and flavour substance shifts during fermentation of Yangjiang douchi. Food Chem..

[35] Amino Y., Takino Y., Kaneko M., Ookura F., Yamamoto M., Kashiwagi T., Iwasaki K. (2016). Synthesis, Characterization, and Evaluation of Thiazolidine Derivatives of Cysteine for Suppressing Eumelanin Production. Chem. Pharm. Bull..

[36] Johnson C.H., Ivanisevic J., Siuzdak G. (2016). Metabolomics: Beyond biomarkers and towards mechanisms. Nat. Rev. Mol. Cell Biol..

[37] Seo S.-H., Jo J.K., Kim E.-J., Park S.-E., Shin S.Y., Park K.M., Son H.-S. (2020). Metabolomics Reveals the Alteration of Metabolic Pathway by Alpha-Melanocyte-Stimulating Hormone in B16F10 Melanoma Cells. Molecules.

[38] Kurokawa M., Ohtsu T., Chatani E., Tamura A. (2023). Hyper Thermostability and Liquid-Crystal-Like Properties of Designed a-Helical Peptide Nanofibers. J. Phys. Chem. B.

[39] Ju X., Cheng S., Li H., Xu X., Wang Z., Du M. (2022). Tyrosinase inhibitory effects of the peptides from fish scale with the metal copper ions chelating ability. Food Chem..

[40] Han J.H., Bang J.S., Choi Y.J., Choung S.-Y. (2019). Anti-melanogenic effects of oyster hydrolysate in UVB-irradiated C57BL/6J mice and B16F10 melanoma cells via downregulation of cAMP signaling pathway. J. Ethnopharmacol..

